# Risk factors for occurrence and abundance of *Aedes aegypti* and *Aedes bromeliae* at hotel compounds in Zanzibar

**DOI:** 10.1186/s13071-021-05005-9

**Published:** 2021-10-22

**Authors:** Ayubo Kampango, Peter Furu, Divakara L. Sarath, Khamis A. Haji, Flemming Konradsen, Karin L. Schiøler, Michael Alifrangis, Fatma Saleh, Christopher W. Weldon

**Affiliations:** 1 Sector de Estudos de Vetores, Instituto Nacional de Saúde (INS), Vila de Marracuene, EN1, Parcela N°3943, Província de Maputo, Mozambique; 2 Department of Zoology and Entomology, University of Pretoria (UP), Hatfield, South Africa; 3 Global Health Section, Department of Public Health, University of Copenhagen, Copenhagen, Denmark; 4 South Asian Clinical Toxicology Research Collaboration (SACTRC), Faculty of Medicine, University of Peradeniya, Peradeniya, Sri Lanka; 5 Zanzibar Malaria Elimination Programme (ZAMEP), Unguja Island, Zanzibar, Tanzania; 6 Center for Medical Parasitology, Department of Immunology and Microbiology, University of Copenhagen, Copenhagen, Denmark; 7 Department of Infectious Diseases, Copenhagen University Hospital (Rigshospitalet), Copenhagen, Denmark; 8 Department of Allied Health Sciences, School of Health and Medical Sciences, The State University of Zanzibar, Unguja Island, Zanzibar, Tanzania

**Keywords:** *Aedes* mosquitoes, Arbovirus vectors, Hotels, Solid waste, Zanzibar

## Abstract

**Background:**

A field survey was performed to investigate local environmental factors promoting occurrence and abundance of *Aedes aegypti* and *Ae. bromeliae* mosquitoes at hotel compounds in the south-east coastal region of Zanzibar Island.

**Methods:**

The potential risk factors were determined using generalized linear mixed models. *Aedes* (*Stegomyia*) spp. indices such as container index (CI) and pupae per container (PPC) index were also estimated.

**Results:**

*Aedes aegypti* and *Ae. bromeliae* were the most abundant vector species, accounting for 70.8% of all *Aedes* mosquitoes collected. The highest CI was observed for plastic containers irrespective of the season, whereas the highest PPC was observed for coconut shells and aluminium containers in the rainy and dry seasons, respectively. The risk of *Aedes* mosquito occurrence and abundance were significantly associated with presence of plastic containers, coconut shells, used tyres and steel containers. These were discarded in shaded places, in the open and gardens, or found in plant nurseries.

**Conclusion:**

This study shows that *Aedes* species of global health significance occur at hotel compounds on this part of Zanzibar Island. The occurrence and abundance are sustained by the presence of abundant and poorly managed solid wastes and containers used for gardening tasks. This highlights an urgent need for the adoption of area-wide environmentally sustainable *Aedes* mosquito management interventions that also integrate solid waste management and ornamental plant production practices for reducing the risk of arboviral disease epidemics.

**Graphical Abstract:**

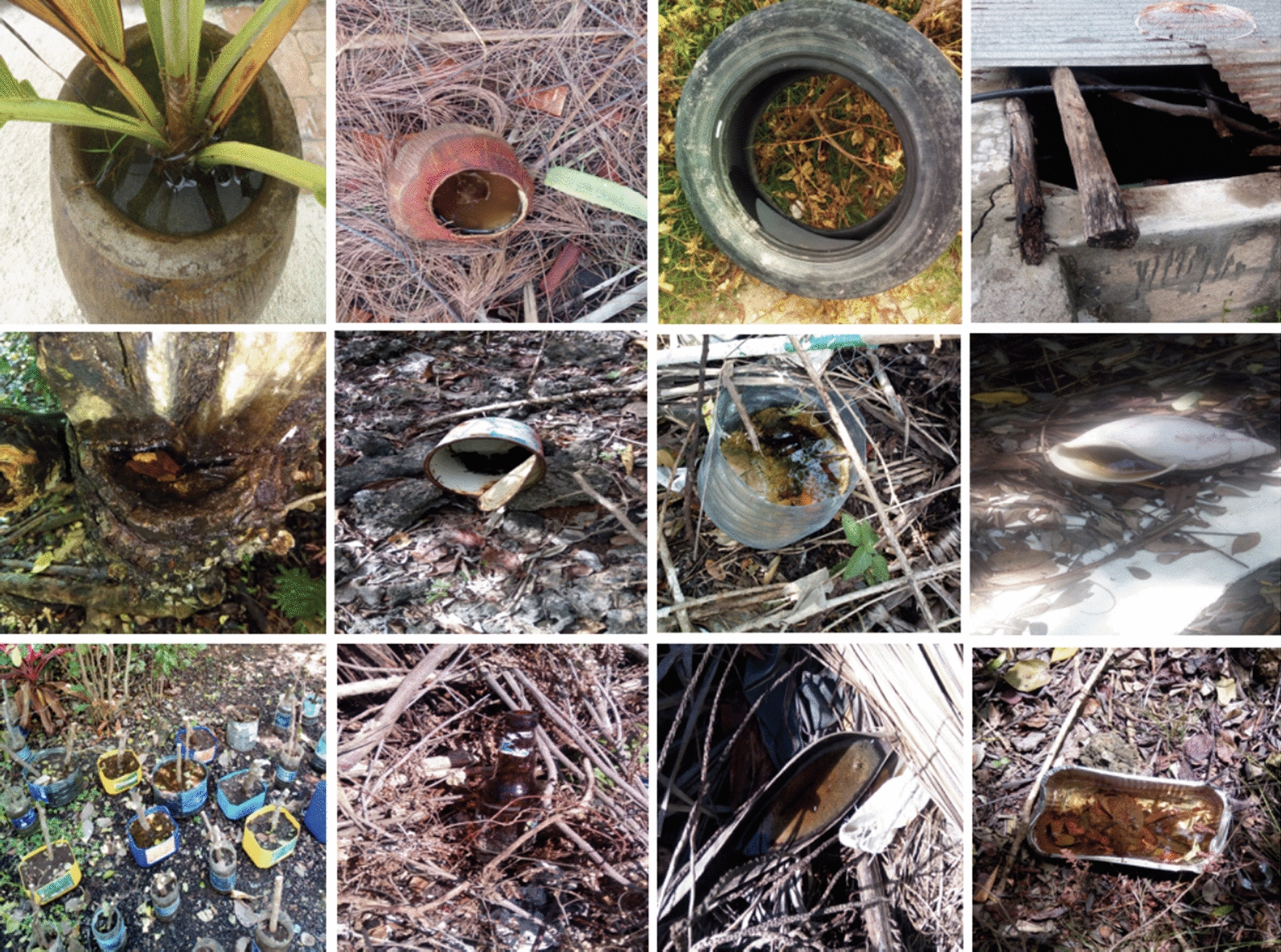

**Supplementary Information:**

The online version contains supplementary material available at 10.1186/s13071-021-05005-9.

## Background

*Aedes* mosquito species include vectors of emerging viruses that present a substantial threat to global health and socio-economic stability [1]. Dengue virus (DENV), yellow fever virus (YFV), chikungunya virus (CHIKV) and Zika virus (ZIKV) are the most notorious *Aedes*-borne viruses due to the severity of illness and magnitude of epidemics that they cause [2]. In recent years, the African continent has reported increasing numbers of DENV, YFV and CHIKV outbreaks [3–5], along with increasing numbers of ZIKV cases in countries such as Angola, Guinea-Bissau, Cabo Verde and Ethiopia [6]. Notably, dengue prevalence in Africa has increased dramatically over the last two decades [5], while yellow fever is showing signs of rebounding as a public health concern after decades of considerable reduction [7–9]. *Aedes aegypti* sensu lato (henceforth *Ae. aegypti*) is the vector implicated in the most explosive outbreaks [10]. However, other *Aedes* species are highly efficient at transmitting life-threatening arboviruses between humans or between other primates and humans. This includes the more poorly investigated native species, such as Ae. *bromeliae*, *Ae. africanus*, *Ae. furcifer*, *Ae. luteocephalus*, *Ae. metallicus*, *Ae. opok*, *Ae. vittatus* and *Ae. simpsoni* sensu stricto [11]. Unprecedented urbanization coupled with poor solid waste management, movement of people and commodities and, not least, climate change are considered main drivers of vector and pathogen spread [4, 12, 13]. Moreover, it is argued that climate change could cause a shift in the burden of mosquito-borne diseases in Africa from malaria to arboviral diseases, as temperature increases may expand the environmental suitability for transmission of dengue and several other vector-borne arboviral diseases on the continent [14].

Mainland Tanzania has observed a surge in *Aedes*-borne arboviral disease transmission, with six dengue outbreaks recorded within the past 10 years. The most recent outbreak, in 2019, spread from Dar-es-Salaam to Dodoma, Morogoro, Pwani, Singida and Tanga, resulting in 6670 confirmed cases and 13 deaths [15]. Similarly, active co-circulation of dengue and chikungunya viruses has been reported from other regions of the mainland [16–18] and neighbouring countries such as Kenya [19]. Currently, there is no entomological evidence of arboviral disease transmission in the Zanzibar Archipelago. However, recent serological surveys have suggested that dengue virus may be circulating in the local population [20–22]. The occurrence of abundant *Ae. aegypti* and *Ae. bromeliae* across urban and rural areas of Zanzibar Island (also known as Unguja) has also been recently confirmed [23–25]. Infestation of Zanzibar Archipelago by these vectors is a serious threat, as arboviral epidemics will have devastating impacts on the fragile public health system and the tourism-based economy. Tourism contributes 27% of the Zanzibar GDP and 80% of foreign revenue [26]. However, the tourism industry is also the most important generator of poorly managed non-biodegradable solid waste in Zanzibar [27]. Some of these solid wastes can sustain a higher diversity of epidemiologically relevant mosquito taxa [23]. In addition, hotels may concentrate visitors and staff moving to and from different highly endemic regions, which increases the likelihood of contact between local vectors and infectious hosts carrying new variants of mosquito-borne arboviruses. For instance, nearly 722,000 tourists arrived in Zanzibar Archipelago between 2019 and 2020 from mainland Tanzania [28], considered a high-risk pathway for pathogen importation into the archipelago [29, 30]. Introduction of new variants of viruses may cause large and uncontrolled epidemics, as observed in recent outbreaks of CHIKV in Africa and Asia [3, 31]. A similar situation was observed for YFV in Brazil in 2018 [32]. Apart from YFV, there is no widely available, safe and effective vaccine against the main mosquito-borne viruses, nor are there any specific antiviral treatments available for the management of disease cases [2, 10]. Therefore, vector control remains the recommended measure to limit mosquito-borne diseases [10, 33]. In Zanzibar, hotels implement periodic blanket spraying with residual-effect insecticide to reduce mosquito bite exposure. This approach, apart from causing widely known environmental repercussions [34], exerts selective pressure on local vector species to develop resistance to common classes of insecticides, as recently confirmed for *Ae. aegypti* populations found at different hotels in Zanzibar (Kampango et al. [23], unpublished). This indicates that non-chemical and environmentally sustainable control practices targeting mosquito sources should be implemented to reduce the risk of arbovirus infection exposure. The design and implementation of cost-effective environmental control approaches require accurate and thorough characterization of key environmental factors favouring the establishment and maintenance of vector populations in and around hotel settings. Hotels can be ideal locations to test the feasibility and efficacy of novel non-chemical environmental interventions before large-scale implementation as they have well delimited and fully managed environments.

The main goal of this study was to determine local environment-associated risk factors for occurrence and exposure to *Aedes* mosquito vectors found at hotel compounds on the eastern coastal region of Zanzibar Island. Findings from this study will expand the knowledge on the entomological profile of potential arbovirus vectors occurring in Zanzibar. Moreover, they may support the implementation of a comprehensive mosquito surveillance system and integrated arbovirus vector management, since hotels in the region are not currently covered by the public surveillance system and routine vector control campaigns.

## Methods

### Description of study sites

**Fig. 1:**
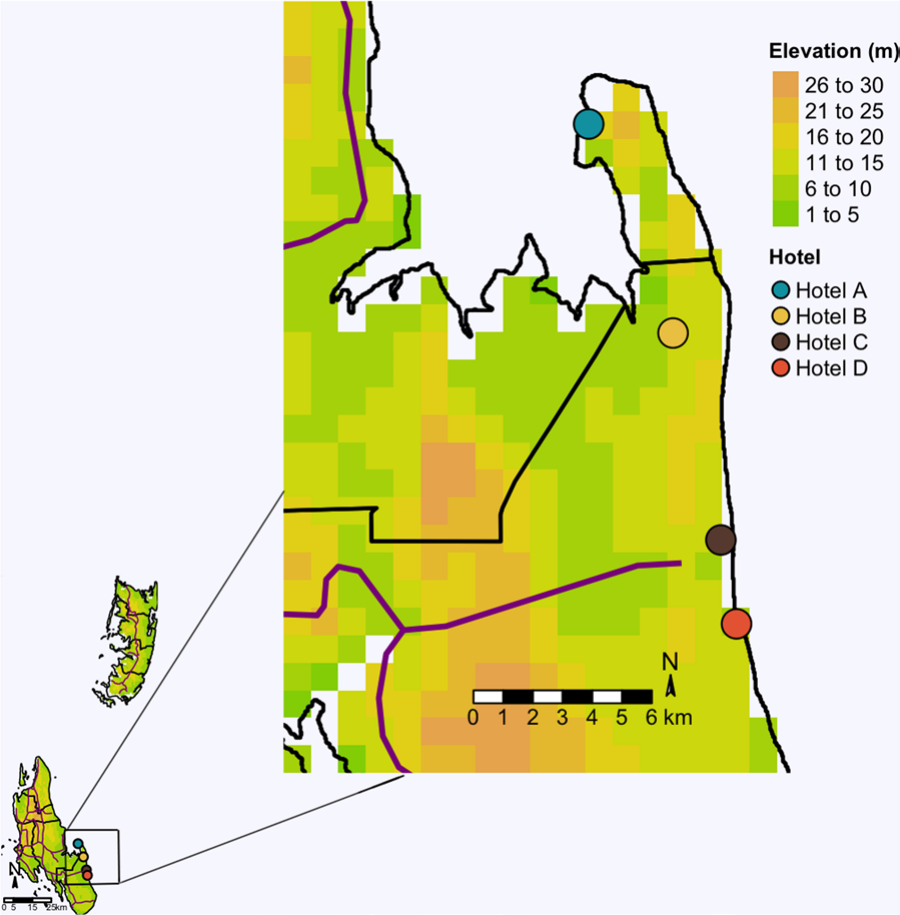
Location of studied hotels on the south-eastern coastal region of Zanzibar Island

The study was carried out at four selected hotels in the south-eastern coastal region of Zanzibar Island (Fig. 1). The hotels, previously described in Kampango et al. [23], were selected according to compound size (total residential and non-residential area not less than one hectare), accessibility by local means of transportation during low and high tourism seasons, willingness to share data and willingness to accept publication of findings. Consent was obtained from hotel management to conduct the study on their properties. For privacy reasons, hotel names are anonymized, and Hotel A, B, C and D used as identifiers. The largest hotel (Hotel B) occupied an area of approximately 28.06 hectares, whilst the remaining hotels encompassed an approximate area of 6.6 hectares (Hotel A), 3.6 hectares (Hotel D), and 2 hectares (Hotel C). The rainfall regime of Zanzibar is divided into two main rainy seasons, the long rainy season (*Masika*) usually extends from mid-March to June, and the short rainy season (*Vuli*) from November to December. Average monthly precipitation ranges from 30 mm in the cold season (July) to 320 mm in the hot season (December) with the accumulated annual rainfall reaching 1600 mm [35]. The relative humidity is usually high, with a monthly average ranging from 87% during the long rainy season (March to June) to 76% in November and December (during the short rainy season), and reaching a minimum of 60% during the dry season (January to March and July to October) [36].

### Mosquito collection

*Aedes* mosquito immature stages were sampled for a period of 10 months from September 2018 to October 2019, encompassing both the rainy and dry seasons. At each hotel, mosquitoes were surveyed over 4 to 6 days in each month using protocols proposed by Tun-Lin et al. [37] and Manrique-Saide et al. [38], with small adaptions as previously detailed in Kampango et al. [23]. For relatively small containers [water volume less than 1 l up to 5 l, e.g., plastic bottles and beer or soda cans (metal containers)], all larvae and pupae were sampled using pipettes. For large containers (water volume > 5 l, e.g., buckets, jerry cans and ceramic pottery) with relatively few specimens, all larvae and pupae were sampled using dippers, sweep nets, or bowls. For the largest containers (water volume > 20 l, e.g., water tanks, wells and septic tanks), specimens were collected by 10–15 random dips/sweeps. If a container was too deep (e.g., wells) samples were collected using small, suspended buckets (approx. 5 l), filling ten buckets from different sites of the water container. Containers without mosquito larvae or pupae were considered as potential larval habitats for any local mosquito species if they had water and physical integrity capable of retaining collected water for at least three consecutive days, the average time required for larvae of several Afrotropical mosquito species to hatch from eggs in the wild [39]. Larval habitats were characterized according to type, function, location, size, sun exposure, presence of vegetation, presence of organic matter and season (Table 1).

**Table 1:** Covariates considered to model the risk factors for *Aedes* mosquito infestation and abundance at hotel compounds in Zanzibar

Habitat characteristics	Category	Description
Type	Plastic containers, Coconut shell, Steel container, Ceramic pot/flowerpot, Glass container, Tree hole, Concrete tank, Fibreglass container, Aluminium foil containers, Mollusc shell, Others	Type of natural or man-made water-holding item, artefact, utensil, etc., found with *Aedes* larvae/pupae or containing water quantity capable of sustaining mosquito immature stages (egg, larva, pupa) for at least 3 days
Location	Garden/open spaces, plant nursery, staff room quarters, solid waste dumpsite/collection area, workshop/laundry, kitchen area, office/administration area, road/pathway, guest quarters, bar/restaurant/lobby, sewage treatment network, other	Geographical space within the hotel compound where mosquito larval habitats have been found
Function	Discarded, gardening, cooking/washing, decoration, well/water collection, construction, AC drainage, septic tank/soak-away, wastewater management, other	Actual daily use or purpose of the item found with mosquito larva or pupa
Size	Small (< 1 m^2^), medium (1–5 m^2^), large (> 5 m^2^)	Estimated size in square metres occupied by larval habitat
Sun exposure	Not exposed, exposed half the day, exposed more than half the day	Amount of time the habitat has been exposed to sunlight
Organic matter^a^	With organic matter, without organic matter	Presence of organic matter debris in the habitat
Vegetation	With vegetation, without vegetation	Presence of floating, submerged, emerged vegetation
Season	Rainy, dry	Rainy season (April–May/June; November and December); dry season (January–March; July–October)

^a^Virtually all habitats found positive for larva/pupae contained visible organic matter. Therefore, this variable was not considered in the risk factor modelling as its association with mosquito was obvious

#### Sample processing and identification

Samples of immature mosquitoes were reared at insectary environmental conditions of 27 ± 2 °C and 75 ± 10% relative humidity until they emerged as adults [40]. Adult mosquitoes were identified morphologically to species level using taxonomic keys for Afrotropical mosquito fauna [11, 41].

### Statistical analysis

#### *Aedes* entomological indices

The following entomological indicators for *Aedes* (*Stegomyia*) spp. were estimated: container index (CI), pupae per container (PPC) and proportion of positive containers (PC). CI was estimated by dividing the total number of positive containers of a given type against the total number of all inspected containers. The PPC was estimated by dividing the total number of pupae collected from a given type of container by the total number of containers inspected, whereas the PC index was estimated as the quotient between the total number of a type of positive containers by the total number of all positive containers found.

#### Modelling the risk factors for *Aedes* mosquitos

Mosquito immature counts had excess zeros as the overall proportion of zero counts was over 30%. The counts were also over-dispersed, showing a positive mean–variance relationship [42]. Therefore, we applied zero-inflated negative binomial mixed models (ZINBM) with log link function, to determine local factors contributing to the abundance of *Ae. aegypti* and *Ae. bromeliae* at study sites, while accounting for excess zeros and overdispersion [43]. Dependent variables were *Ae. aegypti* and *Ae. bromeliae* counts. On the other hand, mixed effects logistic regression was applied to determine the risk factor for presence of any *Aedes* species in general*.* The dependent variable was the presence of *Aedes* immatures (larvae and pupae) (Yes = 1, No = 0). For this case, we assumed that the distribution of presence and absence of *Aedes* immature records followed a binomial distribution with logit function. For ZINBM and mixed effects logistic regression models, the potential risk factors considered were type of container, location, function, size, sun exposure, presence of vegetation and season (Table 1). Organic matter was always present in containers with immature *Aedes*, so it was not included in the model. Hotel and day of survey were considered random factors to account for dependence between repeated measurements across sites over time. A likelihood ratio test comparing the null model (model with dependent variable only) and model with the random factor was applied to determine whether adding a random factor was justifiable. Multicollinearity between predictors was evaluated using the variance inflation factor (VIF). VIF estimates less than or equal to three indicated low collinearity [44]. Variables showing high collinearity were fitted separately with other uncorrelated predictors. Model distributional assumptions were determined by examining model residuals for normality, dispersion and independence (no autocorrelation among residuals). Models were fitted using the package glmmTMB v. 1.0.2.9. [45]. To deal with convergence problems, we combined all underrepresented levels of predictor variables, comprising less than 1% of all level observations, and denoted them as "other". We assumed that these underrepresented levels would introduce more information to the model combined rather than alone. Model diagnostics were performed using the package DHARMa v. 0.3.3.0 [46]. Multicollinearity between covariates was determined using the package performance v.0.6.1 [47]. All data processing tasks and analysis were performed via the software R v. 4.2.0 [48].

## Results

### *Aedes* populations and larval habitats

A total of 1466 water-holding containers were inspected out of which 763 were found positive for *Aedes* mosquitoes (larvae and pupae). A total of 19,533 *Aedes* mosquitoes were collected, comprising 16,187 larvae and 3346 pupae. Of these, 83% (16,207/19,533) and 15.6% (3043/19,533) were *Ae.* (*Stegomyia*) *aegypti* and *Ae.* (*Stegomyia*) *bromeliae*, respectively. Other less frequent species, including *Ae.* (*Stegomyia*) *vittatus*, *Ae.* (*Stegomyia*) *africanus*, *Ae.* (*Stegomyia*) *metallicus*, *Ae.* (*Stegomyia*) *unilineatus*, *Ae.* (*Stegomyia*) *calceatus*, *Ae.* (*Stegomyia*) *heischi* and *Ae.* (*Pseudarmigeres*) *natalensis*, accounted for 1.4% (283/19,533) of all specimens sampled (Table 2 and Additional file 1: Table S1).

**Table 2:** Composition and relative productivity of larval habitats found positive for *Aedes* mosquitos at hotel compound on Zanzibar Island

Season	Type of container	Number of containers		*Stegomyia* larva index			*Aedes* abundance				
		Inspected (%)	Positive (%)	CI	PC	PPC	*Aedes* larva	*Aedes* pupa	*Ae. aegypti*	*Ae. bromeliae*	Other *Aedes*
Rainy	Plastic container	571 (70.1)	372 (69.9)	0.46	0.70	2.88	8551	1644	8319	1803	73
	Coconut shell	56 (6.9)	38 (7.1)	0.05	0.07	5.38	1260	301	1497	64	0
	Used tyre	30 (3.7)	24 (4.5)	0.03	0.05	3.90	665	117	772	10	0
	Steel container	33 (4.1)	25 (4.7)	0.03	0.05	5.42	547	179	669	57	0
	Ceramic pot/flowerpot	19 (2.3)	17 (3.2)	0.02	0.03	2.42	220	46	235	30	1
	Glass container	23 (2.8)	13 (2.4)	0.02	0.02	0.35	191	8	165	34	0
	Tree hole	5 (0.6)	4 (0.8)	0.00	0.01	2.60	72	13	84	1	0
	Concrete tank	31 (3.8)	14 (2.6)	0.02	0.03	0.00	50	0	50	0	0
	Aluminium foil container	5 (0.6)	2 (0.4)	0.002	0.004	0.00	62	0	35	23	4
	Mollusc shell	5 (0.6)	4 (0.8)	0.005	0.01	0.80	22	4	23	3	0
	Other	36 (4.4)	19 (3.6)	0.02	0.04	1.19	182	43	193	32	0
Dry	Plastic container	455 (69.8)	165 (71.4)	0.25	0.51	1.80	3580	819	3303	935	161
	Coconut shell	21 (3.2)	8 (3.5)	0.01	0.02	0.24	15	5	20	0	0
	Used tyre	10 (1.5)	4 (1.7)	0.01	0.01	5.10	143	51	194	0	0
	Steel container	31 (4.8)	10 (4.3)	0.02	0.03	0.87	183	27	143	46	21
	Ceramic pot/flowerpot	12 (1.8)	4 (1.7)	0.01	0.01	0.08	14	1	14	1	0
	Glass container	23 (3.5)	1 (0.4)	0.002	0.00	0.00	15	0	0	0	15
	Tree hole	10 (1.5)	5 (2.2)	0.01	0.02	0.00	54	0	45	1	8
	Concrete tank	48 (7.4)	22 (9.5)	0.03	0.07	0.44	54	21	75	0	0
	Fibreglass container	4 (0.6)	3 (1.3)	0.005	0.01	0.25	87	1	88	0	0
	Aluminium foil container	1 (0.2)	1 (0.4)	0.002	0.003	10.00	9	10	19	0	0
	Mollusc shell	2 (0.3)									
	Other	35 (5.4)	8 (3.5)	0.01		1.60	211	56	264	3	0

*CI* container index, *PC* proportion of positive containers, *PPC* pupae per container

**Fig. 2:**
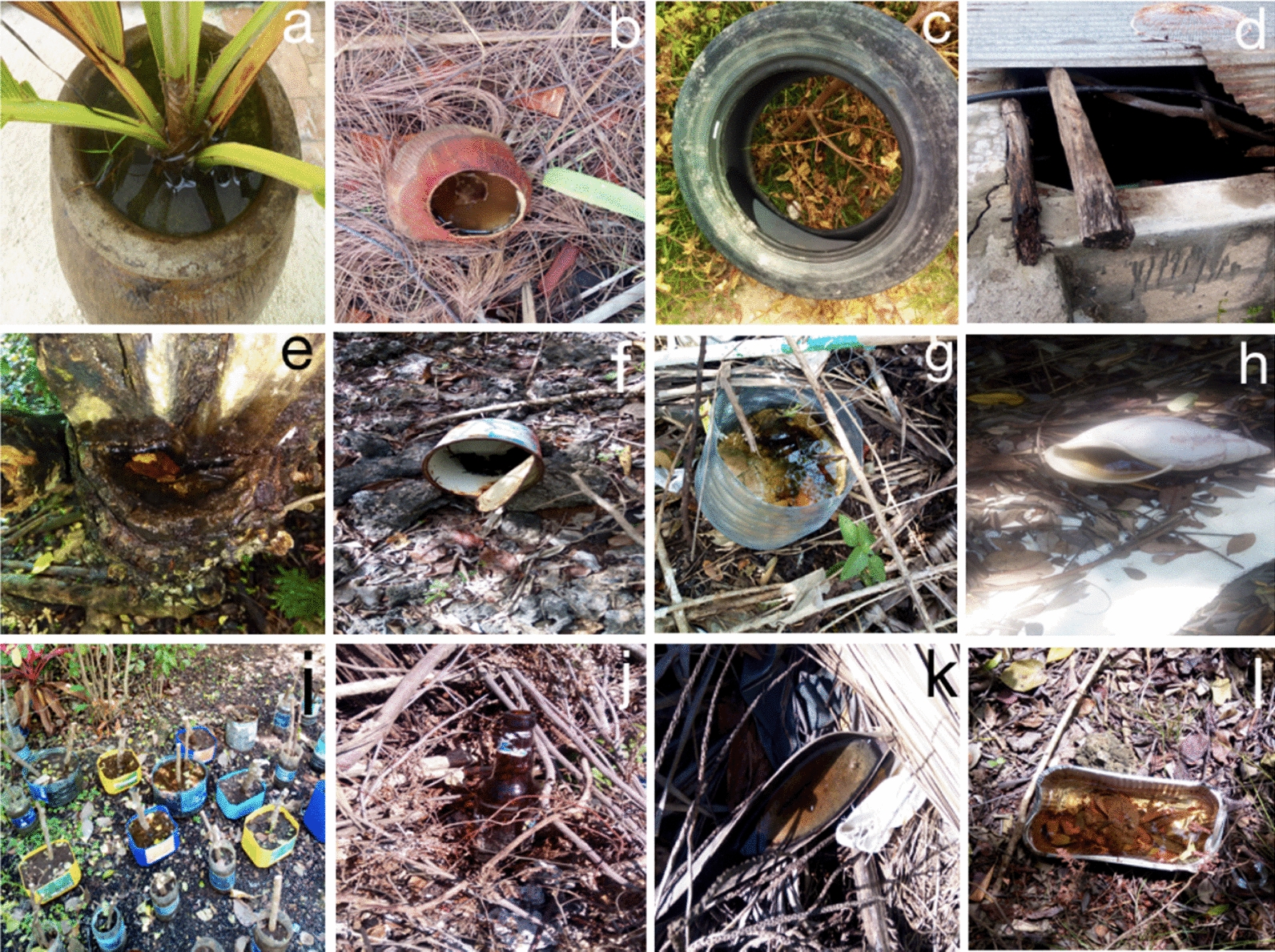
Samples of some common types of larval habitats found with *Aedes* mosquitoes at hotel compounds on the south-eastern coastal region of Zanzibar Island. Flowerpot (**a**); coconut shell (**b**); discarded tyre (**c**); cement tank (**d**); tree hollow (**e**); food can (**f**); water bottle (**g**); mollusc shell (**h**); cut jerry cans and water bottles with rooting plants (**i**); glass bottle (**j**); coconut flower spathe (**k**); aluminium container (**l**)

Plastic containers (mostly bottles and yoghurt cups) were by far the most frequent type of habitat found with larvae and/or pupae. They constituted 69.9% (372/532) and 71.4% (165/231) of all positive habitats recorded in the rainy and dry seasons, respectively, followed by coconut shells, with 7.4% (38/532) versus 3.4% (8/231), metal containers at 4.7% (25/532) versus 4.3% (10/231), and used tyres at 4.5% (24/532) versus 1.7% (4/231). There was also a group of underrepresented larval habitats, such as coconut flower spathes, broken appliances and bottle caps, denoted as "other", which made up nearly 3.6% (19/532) versus 3.5% (8/231) of all habitat types (Table 2, Fig. 2). The highest relative CI was found for plastic containers irrespective of the season. The density of PPC was highest for steel containers (PPC = 5.42) and coconut shells (PPC = 5.38) in the rainy season. In the dry season, the highest PPC was observed for aluminium containers (PPC = 10.0) and used tyres (PPC = 5.10) (Table 2). Nearly 83.4% (1223/1466) of all water-holding containers inspected were discarded items, followed by containers used for gardening activities (5.3%; 78/1466), such as cut jerry cans and plastic bottles for plant propagation (Additional file 1: Table S1 and Fig. 2). Containers were mostly found in gardens or open spaces (62.1%; 909/1466), staff quarters (11.6%; 170/1466), plant nurseries (7.6%; 112/1466) and solid waste dumpsite areas (4.2%; 62/1466) (Additional file 1: Table S1).

### Risk factors for occurrence of *Aedes* mosquitoes

**Fig. 3:**
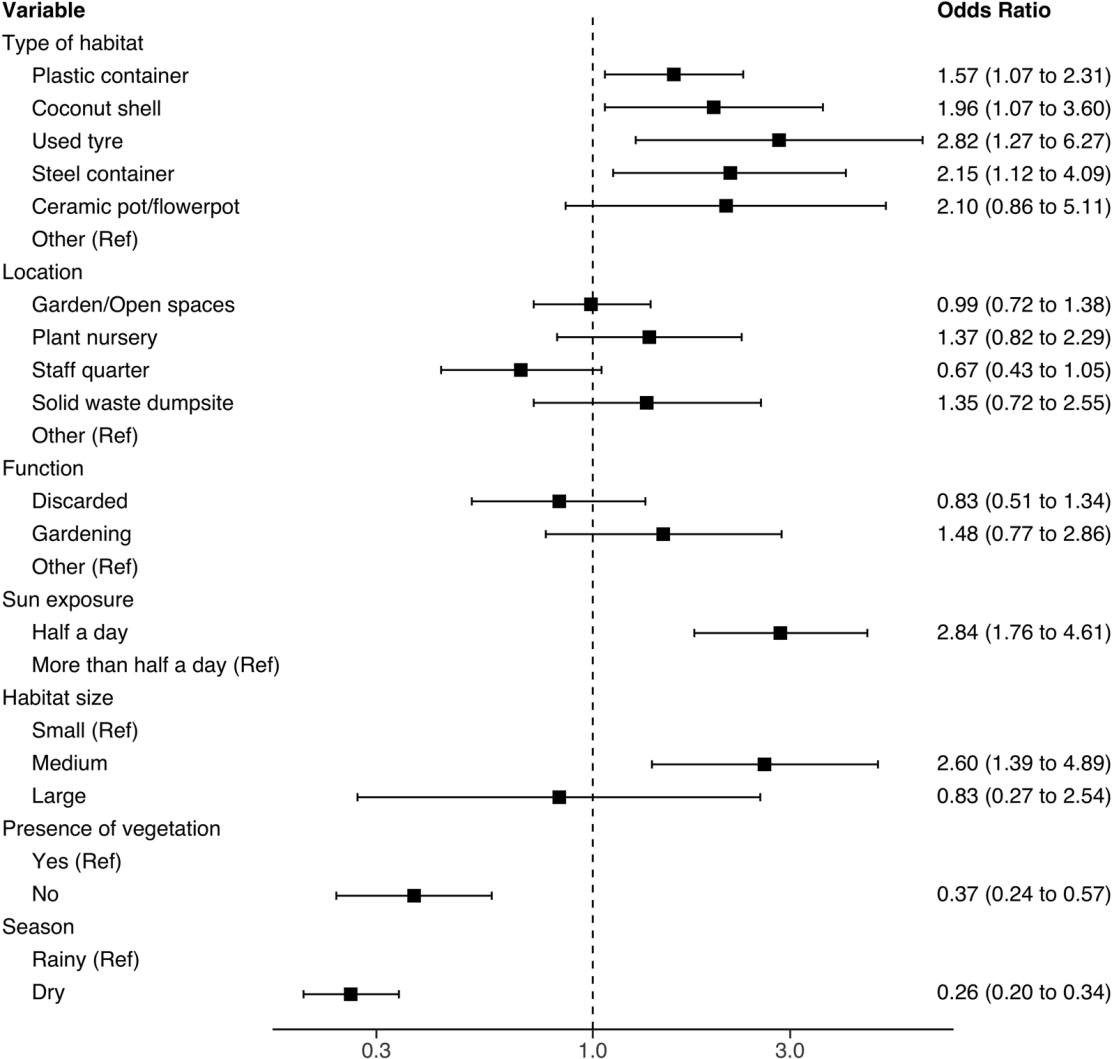
Identified risk factors for occurrence of *Aedes* mosquitoes at hotel compounds on the south-eastern coastal region of Zanzibar Island

The mixed effects logistic regression model indicated that natural and artificial containers such as used tyres, metal containers (e.g., soda cans), plastic containers (e.g., water bottles, jerry cans) and coconut shells were more likely to harbour at least one immature stage of any of the identified *Aedes* mosquitoes compared to other types of water-holding containers (Fig. 3). The likelihood of immature mosquito occurrence was not statistically different between locations within the compounds. It also did not differ according to container function or purpose (Fig. 3). Additionally, containers of medium size and containers that were less exposed to sunlight were less likely to be infested than larger containers or those receiving sunlight for more than half the day (Fig. 3).

### Risk factors for abundance of *Ae. aegypti *and *Ae. bromeliae*

**Fig. 4:**
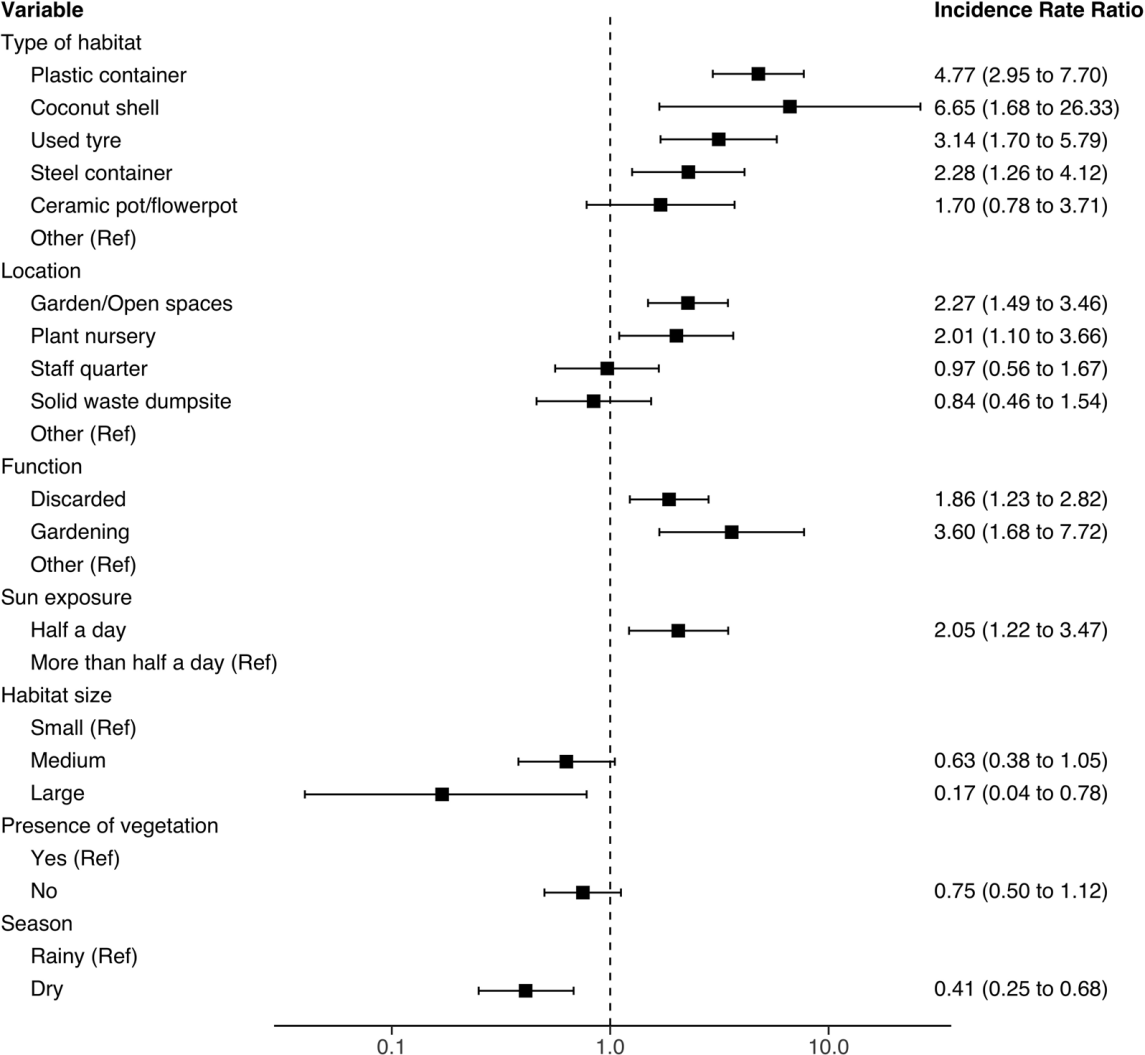
Summary of risk factors for *Ae. aegypti* abundance at hotels compounds on the south-eastern coastal region of Zanzibar Island

**Fig. 5:**
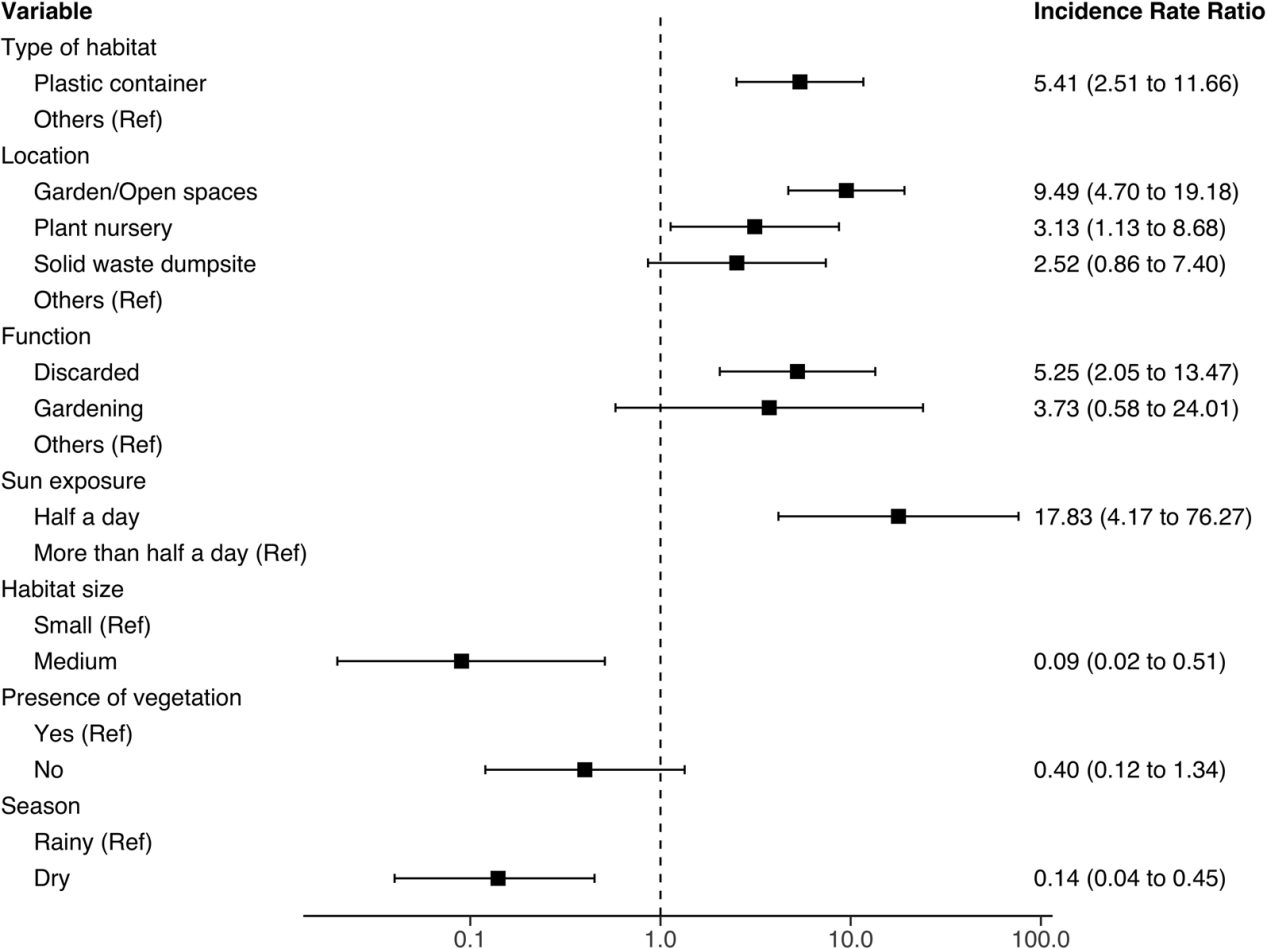
Summary of risk factors for *Ae. bromeliae* abundance at hotels compounds on the south-eastern coastal region of Zanzibar Island

Figure 4 indicates that abundance of *Ae. aegypti* was, in order of magnitude, significantly associated with coconut shells, plastic containers, used tyres and metal containers when compared with other types of containers. The abundance was significantly higher at plant nurseries and garden/open spaces compared to other locations inside the premises. Larval habitats associated with the highest *Ae. aegypti* abundance were mostly discarded items and containers used in gardening tasks and exposed to sunlight for less than half of the day (Fig. 4). Habitats with and without vegetation produced statistically comparable mosquito abundances. In contrast, medium- and large-sized containers and the dry season were associated with lower abundance of *Ae. aegypti* (Fig. 4). Equally, *Ae. bromeliae* was highly abundant in discarded plastic containers found in gardens/open spaces and plant nurseries, and those exposed to sunlight for nearly half of the day (Fig. 5).

## Discussion

Understanding the contribution of environmental factors promoting occurrence and abundance of *Aedes* mosquitoes across space and time is pivotal for the design of cost-effective sustainable vector control interventions. This could involve raising local awareness about solid waste disposal to reduce the risk of mosquito-borne disease exposure and epidemics. Our data clearly indicate that some hotel compounds in Zanzibar provide suitable environmental conditions for establishment of *Ae. aegypti* and *Ae. bromeliae*, and other less frequent *Aedes* mosquito vector species such as *Ae. vittatus*, *Ae. africanus* and *Ae. metallicus*. We found that occurrence and abundance of these vector species are significantly associated with the presence of improperly managed natural and artificial solid wastes and ornamental containers. A great number of studies have reported strong associations between the abundance of *Aedes* mosquitoes and their larval habitats and poorly managed disposables as well as decorative containers [49–51]. These materials can hold water and organic debris for long periods, providing relatively stable micro-environmental and microclimatic conditions for proliferation of diverse mosquito species [24, 25, 51, 52]. We also found high productivity of *Aedes* mosquitoes, particularly *Ae. aegypti* and *Ae. bromeliae*, in natural and artificial containers such as coconut shells, plastic containers, steel containers and used tyres. These types of habitats contributed 92.6% and 93.9% of all *Aedes* larvae and pupae collected, respectively. This finding somewhat corroborates that of Saleh et al. [24, 25], who reported higher risk of *Ae. aegypti* exposure associated with plastic containers and used tyres across rural and urban communities in Zanzibar. However, in contrast to Saleh et al. [24, 25], concrete tanks were one of the least productive habitats in our study sites, contributing only 0.64% of all mosquitoes collected.

Plastic containers had among the lowest density of pupae despite being the most common type of larval habitat found at the sites that we sampled. This finding suggests that plastic containers are less suitable for sustaining the development of mosquito larvae to the pupal stage. Some studies of *Ae. aegypti* have also reported lower productivity for plastic than other types of containers [53, 54]. The factors determining *Ae. aegypti* female choice of certain larval habitat types remain poorly delineated. However, visual stimuli may play an important role, as females of *Ae. aegypti* are more attracted to lower reflectance colour targets [55]. As such, the likelihood of detecting *Ae. aegypti* immatures in darker-coloured containers may be high compared to lighter-coloured containers. Moreover, variation of physicochemical characteristics of the contained water can also affect mosquito detection, as these factors influence both development and survival of mosquitoes at earlier stages. For instance, it has been shown that the average diurnal water temperature in plastic containers can reach up to 35.6 ºC, exceeding the thermal optima range of 15–35 ºC for the development of *Ae. aegypti* immatures [56, 57]. This suggests that plastic containers may become unsuitable larval sites depending on their daily amount of sunlight exposure. On the other hand, higher levels of pH and salinity may also be limiting factors [58]. However, Saleh et al. [24] found no clear association between variations of physicochemical factors and presence of *Ae. aegypti* in Zanzibar City. Further studies on ecological and physiological determinants of the occurrence and abundance of *Aedes* mosquito immature stages in larval habitats are encouraged.

Mixed effects logistic regression and zero-inflated negative binomial mixed models revealed used tyres, plastic containers, coconut shells and metal containers (e.g., beer, soda and food cans) as the types of containers most associated with higher risk of *Ae. aegypti* and *Ae. bromeliae* presence and abundance, respectively. However, the likelihood of infestation was comparable between locations and type of container function or purpose. In contrast to the risk of infestation, the overall abundance of *Aedes* mosquitoes was up to nine times as high (e.g., *An. bromeliae*) in gardens/open spaces and plant nurseries compared to other locations. This finding suggests that these locations should be prioritized when implementing mosquito control interventions. Moreover, the result also indicates that mosquito management practices should be incorporated into ornamental plant production and trading activities, as they may contribute tremendously to maintenance and rapid dissemination of native and invasive vector species throughout the archipelago. Notably, invasive vector species such as *Ae. albopictus* have been successfully introduced and spread worldwide by the trade of ornamental flowers [59, 60]. Higher mosquito abundance was consistently associated with discarded materials and containers used in gardening activities than for other purposes and/or functions. Similar findings were reported for community settings in Zanzibar [25] and Argentina [53]. Furthermore, containers less exposed to sunlight, usually found in densely vegetated places, were more likely to be infested and to have higher mosquito abundance. Similar findings have been reported in other parts of Zanzibar and elsewhere, suggesting that targeted environmental manipulation of shaded places should be prioritized in efforts to control *Aedes* mosquitoes. Virtually all containers found positive for either *Aedes* larvae or pupae contained organic matter detritus. It is well documented that dissolved organic matter provides conditions for development of microorganisms that can act as food for mosquito larvae. However, most recent evidence also shows that dissolved organic matter plays a key role in protecting mosquito immature stages from the lethal effects of solar ultraviolet radiation [61].

The association between poorly managed solid waste and high risk of *Aedes* infestation and abundance in public [24, 25] and private settings (this study) provides evidence for the design and implementation of sustainable environmental mosquito control practices. This includes integrated and consistent solid waste management strategies, periodic inspection and substitution and drainage of ornamental plant water, and periodic waste removal campaigns, so as to reduce the risk of *Aedes*-borne arbovirus epidemics. Taking these actions is essential given recent evidence for resistance in *Ae. aegypti* from Zanzibar to some classes of insecticides approved for use in public health (Kampango et al., in progress). Additionally, this finding is also a call for implementation of strong legislation and regulation leading to environmentally safe waste disposal and treatment. This study was carried out in a relatively small number of hotels, which prevents us from making broader generalizations. Similar types of investigations involving hotels in other eco-geographical regions of Zanzibar may provide a more comprehensive understanding of risks factors for mosquito-borne arbovirus exposure at hotels in the archipelago.

## Conclusion

We conclude that unmanaged solid waste from artificial and natural sources is the most important risk factor for the occurrence and abundance of the arbovirus vectors *Ae. aegypti* and *Ae. bromeliae* at hotel compounds investigated in this study. Notably, the most important sources of *Aedes* mosquitoes are disposable plastic containers (water bottles, yoghurt cups, jerry cans, etc.), used tyres and metal containers (e.g., soda, beer and food cans), and natural products such as coconut shells. Future vector control interventions should target these types of larval habitats, especially in shaded places in garden areas, plant nurseries and in the surrounding communities. Findings from this study underscore the need for development and implementation of area-wide mosquito control interventions that incorporate waste management practices, vegetation manipulation and community mobilization for environmental sanitation. Hotels present a unique opportunity to test the cost-effectiveness of these integrated control approaches, given the human resources, the financial incentives and structured/systematic management of the compounds. We propose that future eco-certification/classification of tourism facilities could include implementation of area-wide non-chemical environmental management practices to reduce mosquito proliferation as an indicator, since mosquito infestation rates remain high in spite of periodic mosquito control activities involving the use of chemical insecticides.

## Supplementary Information

**Additional file 1: Table S1.** Relative abundance of *Aedes* mosquitos found at hotel compounds stratified according to larval habitat characteristics, that is, type of habitat, location, function, size, presence of vegetation, presence of organic matter and season.

## Data Availability

The data that support the findings of this study are available from the corresponding author upon reasonable request.
