# Supplementary material for: Risk factors for occurrence and abundance of Aedes aegypti and Aedes bromeliae at hotel compounds in Zanzibar
Source: Parasit Vectors. 2021 Oct 22;14:544. doi: 10.1186/s13071-021-05005-9 (PMC8539800; doi:10.1186/s13071-021-05005-9)
Supplement: Supplementary file 1 — Additional file 1: Table S1. Relative abundance of Aedes mosquitos found at hotel compounds stratified according to larval habitat characteristics, that is, type of habitat, location, function, size, presence of vegetation, presence of organic matter and season. [file 13071_2021_5005_MOESM1_ESM.docx]

**Supplementary information for**

**Risk factors for occurrence and abundance of *Aedes aegypti* and *Aedes bromeliae* at hotel compounds in Zanzibar**

Ayubo Kampango^1,2^, Peter Furu^3^, Divakara Lional Sarath^4^, Khamis Aimer Haji^5^, Flemming Konradsen^3^, Karin Linda Schiøler^3^, Michael Alifrangis^6,7^, Fatma Saleh^8^, Christopher W. Weldon^2^

^1^Sector de Estudos de Vetores, Instituto Nacional de Saúde (INS), Vila de Marracuene, Província de Maputo, Mozambique

^2^Department of Zoology and Entomology, University of Pretoria (UP), Hatfield, South Africa

^3^Global Health Section, Department of Public Health, University of Copenhagen, Copenhagen, Denmark

^4^South Asian Clinical Toxicology Research Collaboration (SACTRC), Faculty of Medicine, University of Peradeniya, Sri Lanka

^5^Zanzibar Malaria Elimination Programme (ZAMEP), Unguja Island, Zanzibar, Tanzania

^6^Center for Medical Parasitology, Department of Immunology and Microbiology, University of Copenhagen, Copenhagen, Denmark

^7^Department of Infectious Diseases, Copenhagen University Hospital (Rigshospitalet), Copenhagen, Denmark

^8^Department of Allied Health Sciences, School of Health and Medical Sciences, The State University of Zanzibar, Tanzania

Ayubo Kampango ([akampango@gmail.com](mailto:akampango@gmail.com))

Peter Furu ([furu@sund.ku.dk](mailto:furu@sund.ku.dk))

Divakara Lional Sarath ([sarathlional@gmail.com](mailto:sarathlional@gmail.com))

Khamis Ameir Haji ([kahajim@yahoo.com](mailto:kahajim@yahoo.com))

Flemming Konradsen ([flko@sund.ku.dk](mailto:flko@sund.ku.dk))

Karin L. Schiøler ([ksch@sund.ku.dk](mailto:ksch@sund.ku.dk))

Michael Alifrangis ([micali@sund.ku.dk](mailto:micali@sund.ku.dk))

Fatma Saleh ([fatmahamidsaleh@gmail.com](mailto:fatmahamidsaleh@gmail.com))

Christopher W. Weldon ([cwweldon@zoology.up.ac.za](mailto:cwweldon@zoology.up.ac.za))

**Table S1**. Relative abundance of Aedes mosquito found at hotel compounds stratified according to larval habitat characteristics, that is, type of habitat, location, function, size, presence of vegetation, presence of organic matter and season.

| **Variable** | **Larva habitats** | |  | ***Aedes* mosquito abundance** | | | | | |
| --- | --- | --- | --- | --- | --- | --- | --- | --- | --- |
|  | **Inspected (%)** | **Positive (%)** |  | **Total larva (%)** | **Total pupa (%)** | **Total mosquito**  **(%)*** | ***Aedes aegypti* (%)** | ***Aedes bromeliae* (%)** | **Other *Aedes* species (%)** |
| **Type of habitat** |  |  |  |  |  |  |  |  |  |
| Plastic container | 1026 (69.99) | 537 (70.38) |  | 12131 (62.11) | 2463 (73.61) | 14594 (63.81) | 11622 (71.71) | 2738 (89.98) | 234 (82.69) |
| Coconut shell | 77 (5.25) | 46 (6.03) |  | 1275 (6.53) | 306 (9.15) | 1581 (6.91) | 1517 (9.36) | 64 (2.1) | 0 (0) |
| Used tyre | 40 (2.73) | 28 (3.67) |  | 808 (4.14) | 168 (5.02) | 976 (4.27) | 966 (5.96) | 10 (0.33) | 0 (0) |
| Steel container | 64 (4.37) | 35 (4.59) |  | 730 (3.74) | 206 (6.16) | 936 (4.09) | 812 (5.01) | 103 (3.38) | 21 (7.42) |
| Ceramic pot/flowerpot | 31 (2.11) | 21 (2.75) |  | 234 (1.2) | 47 (1.4) | 281 (1.23) | 249 (1.54) | 31 (1.02) | 1 (0.35) |
| Glass container | 46 (3.14) | 14 (1.83) |  | 206 (1.05) | 8 (0.24) | 214 (0.94) | 165 (1.02) | 34 (1.12) | 15 (5.3) |
| Tree hole | 15 (1.02) | 9 (1.18) |  | 126 (0.65) | 13 (0.39) | 139 (0.61) | 129 (0.8) | 2 (0.07) | 8 (2.83) |
| Concrete tank | 79 (5.39) | 36 (4.72) |  | 104 (0.53) | 21 (0.63) | 125 (0.55) | 125 (0.77) | 0 (0) | 0 (0) |
| Fibre glass container | 4 (0.27) | 3 (0.39) |  | 87 (0.45) | 1 (0.03) | 88 (0.38) | 88 (0.54) | 0 (0) | 0 (0) |
| Aluminium foil container | 6 (0.41) | 3 (0.39) |  | 71 (0.36) | 10 (0.3) | 81 (0.35) | 54 (0.33) | 23 (0.76) | 4 (1.41) |
| Mollusc shell | 7 (0.48) | 4 (0.52) |  | 22 (0.11) | 4 (0.12) | 26 (0.11) | 23 (0.14) | 3 (0.1) | 0 (0) |
| Other | 71 (4.84) | 27 (3.54) |  | 393 (2.01) | 99 (2.96) | 492 (2.15) | 457 (2.82) | 35 (1.15) | 0 (0) |
| **Location** |  |  |  |  |  |  |  |  |  |
| Garden/Open spaces | 909 (62.01) | 453 (59.37) |  | 12140 (62.15) | 2123 (63.45) | 12140 (53.08) | 9461 (58.38) | 2459 (80.81) | 220 (77.74) |
| Plant nursery | 112 (7.64) | 74 (9.7) |  | 2360 (12.08) | 490 (14.64) | 2360 (10.32) | 2083 (12.85) | 240 (7.89) | 37 (13.07) |
| Staff quarter | 170 (11.6) | 81 (10.62) |  | 1574 (8.06) | 184 (5.5) | 1574 (6.88) | 1505 (9.29) | 63 (2.07) | 6 (2.12) |
| Solid waste management | 62 (4.23) | 42 (5.5) |  | 704 (3.6) | 116 (3.47) | 704 (3.08) | 596 (3.68) | 108 (3.55) | 0 (0) |
| Workshop/laundry area | 42 (2.86) | 19 (2.49) |  | 699 (3.58) | 137 (4.09) | 699 (3.06) | 601 (3.71) | 97 (3.19) | 1 (0.35) |
| Kitchen area | 25 (1.71) | 14 (1.83) |  | 468 (2.4) | 45 (1.34) | 468 (2.05) | 449 (2.77) | 13 (0.43) | 6 (2.12) |
| Office/Administration | 15 (1.02) | 10 (1.31) |  | 446 (2.28) | 52 (1.55) | 446 (1.95) | 433 (2.67) | 10 (0.33) | 3 (1.06) |
| Road/Street/Pathway | 37 (2.52) | 23 (3.01) |  | 378 (1.94) | 84 (2.51) | 378 (1.65) | 346 (2.13) | 26 (0.85) | 6 (2.12) |
| Guest room area | 43 (2.93) | 23 (3.01) |  | 342 (1.75) | 75 (2.24) | 342 (1.5) | 336 (2.07) | 2 (0.07) | 4 (1.41) |
| Bar/Restaurante area | 6 (0.41) | 3 (0.39) |  | 82 (0.42) | 5 (0.15) | 82 (0.36) | 82 (0.51) | 0 (0) | 0 (0) |
| Sewage treatment network | 6 (0.41) | 4 (0.52) |  | 6 (0.03) | 0 (0) | 6 (0.03) | 5 (0.03) | 1 (0.03) | 0 (0) |
| Other | 39 (2.66) | 17 (2.23) |  | 334 (1.71) | 35 (1.05) | 334 (1.46) | 310 (1.91) | 24 (0.79) | 0 (0) |
| **Function** |  |  |  |  |  |  |  |  |  |
| Discarded | 1223 (83.42) | 628 (82.31) |  | 16220 (83.04) | 2515 (75.16) | 16220 (70.92) | 13083 (80.72) | 2863 (94.08) | 274 (96.82) |
| Gardening | 78 (5.32) | 51 (6.68) |  | 2063 (10.56) | 622 (18.59) | 2063 (9.02) | 1950 (12.03) | 105 (3.45) | 8 (2.83) |
| Cooking/Washing | 19 (1.3) | 10 (1.31) |  | 133 (0.68) | 12 (0.36) | 133 (0.58) | 116 (0.72) | 17 (0.56) | 0 (0) |
| Decoration | 6 (0.41) | 5 (0.66) |  | 119 (0.61) | 33 (0.99) | 119 (0.52) | 117 (0.72) | 1 (0.03) | 1 (0.35) |
| Well/Rain water collection | 19 (1.3) | 9 (1.18) |  | 40 (0.2) | 2 (0.06) | 40 (0.17) | 40 (0.25) | 0 (0) | 0 (0) |
| Construction | 6 (0.41) | 3 (0.39) |  | 25 (0.13) | 2 (0.06) | 25 (0.11) | 25 (0.15) | 0 (0) | 0 (0) |
| AC drainage | 6 (0.41) | 1 (0.13) |  | 30 (0.15) | 0 (0) | 30 (0.13) | 30 (0.19) | 0 (0) | 0 (0) |
| Septic tank/Soak away | 40 (2.73) | 17 (2.23) |  | 26 (0.13) | 0 (0) | 26 (0.11) | 26 (0.16) | 0 (0) | 0 (0) |
| Wastewater management | 4 (0.27) | 2 (0.26) |  | 29 (0.15) | 19 (0.57) | 29 (0.13) | 29 (0.18) | 0 (0) | 0 (0) |
| Other | 65 (4.43) | 37 (4.85) |  | 848 (4.34) | 141 (4.21) | 848 (3.71) | 791 (4.88) | 57 (1.87) | 0 (0) |
| **Container size** |  |  |  |  |  |  |  |  |  |
| Small (<1m2) | 1365 (93.11) | 711 (93.18) |  | 19277 (98.69) | 3287 (98.24) | 19277 (84.28) | 15973 (98.56) | 3021 (99.28) | 283 (100) |
| Medium (1 - 5m2) | 69 (4.71) | 38 (4.98) |  | 225 (1.15) | 59 (1.76) | 225 (0.98) | 203 (1.25) | 22 (0.72) | 0 (0) |
| Large (>5m) | 32 (2.18) | 14 (1.83) |  | 31 (0.16) | 0 (0) | 31 (0.14) | 31 (0.19) | 0 (0) | 0 (0) |
| **Sun exposure** |  |  |  |  |  |  |  |  |  |
| Half a day | 1320 (90.04) | 718 (94.1) |  | 19050 (97.53) | 3302 (98.68) | 19050 (83.29) | 15738 (97.11) | 3029 (99.54) | 283 (100) |
| More than half a day | 108 (7.37) | 30 (3.93) |  | 421 (2.16) | 44 (1.32) | 421 (1.84) | 409 (2.52) | 12 (0.39) | 0 (0) |
| Not exposed | 38 (2.59) | 15 (1.97) |  | 62 (0.32) | 0 (0) | 62 (0.27) | 60 (0.37) | 2 (0.07) | 0 (0) |
| **Organic matter** |  |  |  |  |  |  |  |  |  |
| With Organic matter | 1459 (99.52) | 763 (100) |  | 19533 (100) | 3346 (100) | 19533 (85.4) | 16207 (100) | 3043 (100) | 283 (100) |
| Without Organic matter | 7 (0.48) | 0 (0) |  | 0 (0) | 0 (0) | 0 (0) | 0 (0) | 0 (0) | 0 (0) |
| **Presence of vegetation** |  |  |  |  |  |  |  |  |  |
| With vegetation | 122 (8.32) | 79 (10.35) |  | 2017 (10.33) | 122 (3.65) | 2017 (8.82) | 1403 (8.66) | 585 (19.22) | 29 (10.25) |
| Without vegetation | 1344 (91.68) | 684 (89.65) |  | 17516 (89.67) | 3224 (96.35) | 17516 (76.58) | 14804 (91.34) | 2458 (80.78) | 254 (89.75) |
| **Season** |  |  |  |  |  |  |  |  |  |
| Rainy | 814 (55.53) | 532 (69.72) |  | 14177 (72.58) | 2355 (70.38) | 14177 (61.98) | 12042 (74.3) | 2057 (67.6) | 78 (27.56) |
| Dry | 652 (44.47) | 231 (30.28) |  | 5356 (27.42) | 991 (29.62) | 5356 (23.42) | 4165 (25.7) | 986 (32.4) | 205 (72.44) |

*Sum of total larva and total pupa collected
